# Continuum of maternity care in Zambia: a national representative survey

**DOI:** 10.1186/s12884-021-04080-1

**Published:** 2021-09-05

**Authors:** Quraish Sserwanja, Milton W. Musaba, Linet M. Mutisya, Emmanuel Olal, David Mukunya

**Affiliations:** 1 Programs Department, GOAL, Arkaweet Block 65 House No. 227, Khartoum, Sudan; 2 Department of Obstetrics and Gynaecology, Busitema University, Mbale, Uganda; 3 Maternal and Child Health Project, Swedish Organization for Global Health, Mayuge, Uganda; 4 Yotkom Medical Centre, Kitgum, Uganda; 5 Department of Community and Public Health, Busitema University, Mbale, Uganda; 6 Sanyu Africa Research Institute, Mbale, Uganda

**Keywords:** Continuum of care, Antenatal care, Postnatal care, Childbirth, Utilization, Women and Zambia

## Abstract

**Background:**

Globally, over half of maternal deaths are related to pregnancy-related complications. Provision of a continuum of care during pregnancy, childbirth and the postnatal period results in reduced maternal and neonatal morbidity and mortality. Hence this study determined the prevalence of the continuum of care and its determinants among women in Zambia.

**Methods:**

We used weighted data from the Zambian Demographic and Health Survey (ZDHS) of 2018 for 7325 women aged 15 to 49 years. Multistage stratified sampling was used to select study participants. Complete continuum of care was considered when a woman had; at least four antenatal care (ANC) contacts, utilized a health facility for childbirth and had at least one postnatal check-up within six weeks. We conducted multivariable logistic regression to explore continuum of care in Zambia. All our analyses were done using SPSS version 25.

**Results:**

Of the 7,325 women, 38.0% (2787/7325) (95% confidence interval (CI): 36.9-39.1) had complete continuum of maternal healthcare. Women who had attained tertiary level of education (adjusted odds ratio (AOR): 1.93, 95% CI: 1.09-3.42) and whose partners had also attained tertiary level of education (AOR: 2.58, 95% CI: 1.54-4.32) were more likely to utilize the whole continuum of care compared to those who had no education. Women who initiated ANC after the first trimester (AOR: 0.46, 95% CI: 0.39-0.53) were less likely to utilize the whole continuum of care compared to those who initiated in the first semester. Women with exposure to radio (AOR: 1.58, 95% CI: 1.27-1.96) were more likely to utilize the whole continuum of care compared to those who were not exposed to radio. Women residing in the Western province were less likely to utilize the entire continuum of care compared to those in the other nine provinces.

**Conclusion:**

Level of education of the women and of their partners, early timing of ANC initiation, residing in other provinces other than the Western province, and exposure to information through radio were positively associated with utilization of the entire continuum of care. Improving literacy levels and promoting maternity services through radio may improve the level of utilization of maternity services.

**Supplementary Information:**

The online version contains supplementary material available at 10.1186/s12884-021-04080-1.

## Background

Globally, at least half of maternal mortality results from pregnancy complications, and over 277,300 (94%) of these deaths occur in low- and middle-income countries and roughly two-thirds (196,000) of the deaths take place in sub-Saharan Africa [1, 2]. A woman’s lifetime risk of maternal death in sub-Saharan Africa is 1 in 59, far higher than the risk in all low-income countries, estimated at 1 in 160 [3]. Majority of these preventable maternal and perinatal deaths occur in the intrapartum and immediate postpartum period [4] and have been linked to behavioral risk factors [5]. The direct maternal deaths and could be prevented by strengthening and improving access to the whole continuum of maternity care [6, 7]. Additionally, 1 in 12 children die before reaching the age of five years and most of these deaths occur in the neonatal period [8, 9]. Neonatal mortality contributes to almost 45% of all under-5 childhood deaths worldwide [8, 9]. Globally, 2.4 million neonates died in 2019 which approximates to 6,700 neonatal deaths daily [10]. Sub-Saharan Africa has the highest neonatal mortality rate estimated at 27 deaths per 1,000 live births [10, 11].

Ensuring accelerated progress towards achieving sustainable development goal (SDG) 3 needs global health focus on programs and policies targeting reduction of preventable maternal, newborn and child mortality and morbidity [4]. Limited access and low utilization of maternal healthcare services are some of the main predictors of negative maternal health outcomes in low income countries such as Zambia [12, 13]. It is estimated that if effective health measures are ensured during childbirth and in the first week of life, we could avert over 80% of maternal deaths and 66% of neonatal deaths globally [4, 14]. To ensure this, cost effective measures within the reach of low and middle income countries such as continuum of care are required [4]. Continuum of care ensures uninterrupted flow in the care at all stages hence improving maternal and child health outcomes [15]. Complete exposure to the continuum of care compared to separate healthcare ensures that utilization at each stage builds on the effect of the previous stage of healthcare access.

Reducing maternal mortality requires a dramatic increase in utilization of health facilities during childbirth and quality of maternal health care delivered [16]. The World Health Organization (WHO) global guidelines recommend that women deliver at facilities with the capacity to manage emergencies [17]. Consistent with WHO guidelines, the Zambian guidelines recommend all women to have childbirth in a health facility [18]. In our study, we focused on the continuity of care over time from pregnancy through childbirth to the postpartum period. We looked at antenatal care (ANC) attendance, health facility utilization during childbirth and postnatal care (PNC).

Adequate, timely and quality ANC has been shown to improve birth preparedness and increase skilled birth attendance [6]. Further, it enables early identification and management of pregnancy related illnesses and complications [6, 19]. Health facility utilization during childbirth increases the chances of a safe, clean environment that is well-equipped with skilled personnel, drugs, and supplies necessary for effective and timely management of childbirth complications [4]. Additionally, PNC enables timely identification of any complications that might affect maternal and neonatal health outcomes [4]. These three interventions are crucial in lowering maternal and neonatal mortality rates, thus the need to provide all of them in a continuum of care [20].

As per the National Health Strategic Plan for 2017–2021, Zambia set a target to decrease the maternal mortality ratio (MMR) from 398 to 162 maternal deaths per 100,000 live births by 2021 [21]. Over the last two decades, Zambia has achieved a steady reduction in the MMR which currently stands at 252 deaths per 100,000 live births [22]. However, this MMR is almost four times higher than the SDG 3 target of 70 deaths per 100,000 live births by 2030 [9]. Most studies have focused on exploring factors associated with use of individual maternal health services, but few have explored the utilization of care as a continuum. To ensure effective continuum of care program designing and implementation, it is crucial to understand the healthcare seeking gaps along the continuum pathway and the factors associated with the gaps. Our study aimed at describing the factors influencing the continuum of care during pregnancy, at childbirth and during postpartum period among women in Zambia. The findings will inform the design of programs and strategies aimed at improving level of utilization of the continuum of care, in order to reduce the maternal and neonatal mortality.

## Methods

### Study design and participants

The Zambian Demographic and Health Survey (ZDHS) was a nationally representative cross-sectional study conducted using validated questionnaires between 18^th^ July 2018 and 24^th^ January 2019 [22]. It is a periodic survey that is carried out every five years as part of the MEASURE DHS global survey and collects information on demographic, health, and nutrition indicators. The survey used stratified two-stage cluster sampling design that resulted in the random selection of a representative sample of 13,625 households [22]. The first stage involved 545 cluster (sample points) selection which consisted of enumeration areas using a sampling frame that was used during the 2010 census of population and housing (CPH) [22]. Zambia is divided into 10 provinces which are further divided into districts, constituencies, and wards [22]. Wards, the smallest administrative units, were further divided during the 2010 CPH into census supervisory areas which were later divided into enumeration areas. Enumeration areas were selected with a probability proportional to their size within each sampling stratum. Systematic sampling was applied to select households in the second stage. A detailed explanation of the sampling process is available in the ZDHS 2018 report [22].

Women aged 15-49 years who were either permanent residents or visitors who had stayed in the selected households the night before the survey were eligible for interviews and a total of 13,683 women being interviewed. Four questionnaires were used in the survey and our study used data that was obtained using validated women’s and household questionnaires. Our secondary analysis included women of reproductive age (15-49 years) who had given birth in the last five years preceding the survey that yielded a weighted sample of 7,325 women. Written informed consent was provided by all participants of the survey. Written permission to access the whole ZDHS database was obtained through DHS program website at this address https://dhsprogram.com/

### Variables

#### Outcome variables

Complete continuum of maternal healthcare is the outcome variable, Complete continuum of care was constructed into binary variable with complete coded as 1 and incomplete coded as 0. Complete continuum of maternal healthcare was considered when a woman reported having had:

1. Had at least four ANC (ANC4+) contacts during the most recent childbirth.
2. Utilized a health facility during the most recent childbirth and
3. Had at least one postnatal check-up within six weeks after childbirth [4, 23]

Continuum of care was considered discontinued if the woman skipped any of the steps above [4, 24, 25]. Additionally, having four or more ANC contacts was considered as continued care during pregnancy while utilizing health facility during childbirth with four or more ANC contacts was considered as continued care during delivery.

### Independent variables

Independent variables were categorized into women, partners’ and household characteristics which were chosen basing on previous studies [26–28] and availability in the ZDHS database.

#### Partner characteristics

This included the partner’s highest level of education (no education, primary, secondary, and tertiary).

#### Household characteristics

Wealth index of household (categorized into quintiles: richest, richer, middle poorer and poorest), type of residence (urban and rural), and province/region that included the official 10 provinces of Zambia. Wealth index is a measure of relative household economic status and was calculated by DHS from information on household asset ownership using principal component analysis [22, 29].

#### Women’s characteristics

Age (15-24, 25-34, and 35-49), level of education (no education, primary, secondary, and tertiary), exposure to newspapers/magazines, radio and TV (yes and no), preceding birth interval ( less than 24 months and 24 months and above), parity (one, two and three or more), age at firth birth (less than 20 and 20 and above), ANC timing (first trimester and second or third trimester), marital status (married and not married), age at first sex (less than 18 and 18 and above), working status (working and not working) and decision making for seeking healthcare (involved and not involved).

### Statistical analysis

We used the SPSS analytic software version 25.0 complex samples package. Weighted data was used to account for the unequal probability sampling in different strata. Frequency distributions were used to describe the background characteristics of the women. Bivariable and multivariable logistic regression analyses were conducted to identify the association among various independent variables at all the three levels of continuum of care. Independent variables found significant at p-value less than 0.25 in the bivariable analysis were included in the multivariable models. We presented two models; the primary model, which adjusted for only factors without missing data, and the secondary model, which included all the factors (with and without missing data) and adjusted for those with missing data. The secondary model included residence, provinces, exposure to mass media, working and marital status, level of education, wealth index, timing of ANC initiation, preceding birth interval, age at first sexual intercourse and first birth and healthcare seeking decision making. Adjusted odds ratios (AOR), 95% confidence intervals (CI) and p-values were calculated with statistical significance level set at p-value < 0.05. Sensitivity analysis was done by analyzing continued care during pregnancy with having eight or more ANC contacts as the outcome.

## Results

The mean age of women was 28.5 (standard deviation (SD) 7.5) years with majority (41.2%) of them being 25-34 years. while that of the mothers was 28.80 (SD 6.83). Majority of the women resided in rural areas (61.6%), were married (75.2%), had primary education (49.1%), initiated ANC after first trimester (62.0%), had their first sexual intercourse when they were less than 18 years (74.3%) and had their first pregnancy when they were below 20 years (68.1%). Of the 7,325 women, 38.0% (2787/7325) (95% CI: 36.9-39.1) had complete continuum of maternity care (four or more ANC contacts, health facility utilization during childbirth and postnatal care attendance). Further analysis showed 64.1% (4651/7262) (95% CI: 63.1-65.2) had four or more ANC contacts (continued care during pregnancy) while 57.3% (4199/7325) (95% CI: 56.2-58.5) had continued care at delivery (four or more ANC contacts and utilized a health facility for childbirth). More detailed characteristics of study participants are shown in Table 1.

**Table 1:** Background characteristics of women who had given birth in the last 5 years

Characteristics	N=7325	%
**Parity**		
Three or more	3884	53.0
Two	1440	19.7
One	2001	27.3
**Residence**		
Urban	2811	38.4
Rural	4513	61.6
**Provinces**		
Central	640	8.7
Copper belt	969	13.2
Eastern	983	13.4
Luapula	640	8.7
Lusaka	1219	16.6
Muchinga	433	5.9
Northern	615	8.4
North Western	404	5.5
Southern	946	12.9
Western	477	6.5
**Working status**		
Not working	3835	52.4
Working	3490	47.6
**Marital status**		
Not Married	1819	24.8
Married	5506	75.2
**Education Level**		
No Education	689	9.4
Primary Education	3595	49.1
Secondary Education	2726	37.2
Tertiary	316	4.3
**Wealth Index**		
Poorest	1676	22.9
Poorer	1527	20.8
Middle	1390	19.0
Richer	1471	20.1
Richest	1262	17.2
**Age in years**		
15-24	2605	35.6
25-34	3020	41.2
35-49	1699	23.2
**ANC timing**^a^		
Second or third trimester	4542	62.0
First trimester	2690	36.7
**Exposure to Newspapers**		
Yes	1217	16.6
No	6108	83.4
**Exposure to Radio**		
Yes	3278	44.8
No	4047	55.2
**Exposure to TV**		
Yes	2581	35.2
No	4744	64.8
**Preceding Birth Interval**^b^		
24 months and above	4666	63.7
Less than 24 months	822	11.2
**Partner’s education**^c^		
No education	322	4.4
Primary	2011	27.5
Secondary	2530	34.5
Tertiary	444	6.1
**Age at first sex**		
Less than 18 years	5444	74.3
18 years and above	1880	25.7
**Healthcare seeking decision**^d^		
Involved	4374	59.7
Not involved	1132	15.5
**Age at first birth**		
Less than 20 years	4987	68.1
20 years and above	2338	31.9
**ANC attendance**^e^		
Less than 4	2611	35.9
4 and above	4651	64.1
**Place of delivery**		
Home	951	13.0
Health Facility	6373	87.0
**Postnatal care**		
No	2691	36.7
Yes	4633	63.2

^a^Missing 93, ^b^missing 1836, ^c^missing 2018, ^d^missing 1819, ^e^missing 63

### Factors associated with complete continuum of maternity care

Results from multivariable logistic regression (Table 2) showed that province, timing of ANC initiation, exposure to radio, partners’ and women’s level of education were associated with complete continuum of maternity.

**Table 2:** Predictors of complete continuum of maternity care in Zambia

Characteristics	Complete continued care n=2787	P-value	Crude model COR (95%CI)	P-value	Primary Model AOR (95% CI)	Secondary Model AOR (95% CI)
**Parity**		0.223		0.408		
Three or more	1445 (51.9)		1			
Two	551(19.7)		1.04 (0.89-1.23)			
One	791 (28.4)		1.10 (0.96-1.27)			
**Residence**		0.003		0.082		
Rural	1656 (59.4)		1		1	1
Urban	1130 (40.6)		1.16 (0.98-1.37)		0.99(0.79-1.26)	1.05 (0.79-1.40)
**Provinces**		<0.001		<0.001		
Western	109 (3.9)		1		1	1
Copperbelt	422 (15.1)		**2.61 (1.81-3.75)**		**2.30 (1.58-3.35)**	**3.11 (1.85-5.23)**
Eastern	400 (14.4)		**2.32 (1.62-3.34)**		**2.22 (1.55-3.19)**	**2.91 (1.79-4.74)**
Luapula	267 (9.6)		**2.41 (1.59-3.67)**		**2.22 (1.49-3.29)**	**2.90 (1.74-4.82)**
Lusaka	488 (17.5)		**2.25 (1.56-3.26)**		**1.95 (1.33-2.87)**	**2.81 (1.67-4.75)**
Muchinga	209 (7.5)		**3.16 (2.09-4.76)**		**3.19 (2.17-4.69)**	**4.08 (2.46-6.75)**
Northern	272 (9.8)		**2.69 (1.61-4.49)**		**2.66 (1.60-4.41)**	**3.63 (1.92-6.85)**
North Western	164 (5.9)		**2.31 (1.62-3.31)**		**2.40 (1.69-3.40)**	**3.88 (2.29-6.57)**
Southern	264 (9.5)		1.31 (0.87-1.97)		1.25 (0.84-1.87)	1.48 (0.91-2.43)
Central	191 (6.9)		1.44 (0.98-2.12)		1.32 (0.90-1.95)	**1.86 (1.15-3.03)**
**Working status**		0.041		0.128		
Not working	1416 (50.8)		1		1	1
Working	1370 (49.2)		1.10 (0.97-1.26)		1.05 (0.93-1.19)	0.89 (0.74-1.06)
**Marital status**		0.006		0.022		
Not Married	643 (23.1)		1		1	-
Married	2143 (76.9)		**1.17 (1.02-1.33)**		1.10 (0.96-1.26)	-
**Education Level**		<0.001		<0.001		
No Education	198 (7.1)		1			1
Primary Education	1316 (47.2)		**1.43 (1.14-1.79)**			**1.29 (1.01-1.64**)
Secondary Education	1091 (39.1)		**1.65 (1.33-2.05)**			1.21 (0.91-1.61)
Tertiary	182 (6.5)		**3.34 (2.32-4.82)**			**1.93 (1.09-3.42)**
**Wealth Index**		<0.001		0.052		
Poorest	611 (21.9)		1		1	1
Poorer	550 (19.7)		0.98 (0.83-1.16)		0.93 (0.78-1.11)	0.88 (0.71-1.10)
Middle	515 (18.5)		1.03 (0.84-1.25)		0.97 (0.79-1.18)	0.91 (0.70-1.18)
Richer	547 (19.6)		1.03 (0.81-1.31)		0.88 (0.65-1.19)	0.94 (0.62-1.43)
Richest	563 (20.2)		**1.40 (1.09-1.81)**		0.92 (0.66-1.30)	0.79 (0.48-1.29)
**Age in years**		0.721		0.777		
35-49	644 (23.1)		1			
25-34	1165 (41.8)		1.03 (0.88-1.20)			
15-24	978 (35.1)		0.98 (0.85-1.14)			
**ANC timing**		<0.001		<0.001		
First trimester	1353 (48.6)		1			1
Second or third trimester	1429 (51.4)		**0.45 (0.40-0.51)**			**0.46 (0.39-0.53)**
**Newspaper Exposure**		<0.001		0.001		
No	2237 (80.3)		1		1	1
Yes	550 (19.7)		**1.43 (1.15-1.77)**		1.09 (0.88-1.35)	1.10 (0.86-1.41)
**Exposure to Radio**		<0.001		<0.001		
No	1347 (48.3)		1		1	1
Yes	1440 (51.7)		**1.57 (1.38-1.79)**		**1.43 (1.23-1.68)**	**1.58 (1.27-1.96)**
**Exposure to TV**		<0.001		0.002		
No	1705 (61.2)		1		1	1
Yes	1082 (38.8)		**1.29 (1.10-1.51)**		0.96 (0.77-1.18)	0.92 (0.73-1.16)
**Preceding Birth Interval**		0.001		0.006		
Less than 24 months	263 (12.9)		1			1
24 months and above	1778 (87.1)		**1.31 (1.08-1.59)**			1.23 (0.99-1.52)
**Partner’s education**		<0.001		<0.001		
No education	93 (4.5)		1			1
Primary	719 (34.5)		1.37 (0.95-1.96)			1.31 (0.88-1.94)
Secondary	1012 (48.6)		**1.64 (1.17-2.29)**			1.40 (0.96-2.05)
Tertiary	258 (12.4)		**3.42 (2.32-5.03)**			**2.58 (1.54-4.32)**
**Age at first sex**		<0.001		0.001		
18 years and above	794 (28.5)		**1**		1	1
Less than 18 years	1993 (71.5)		**0.79 (0.69-0.91)**		1.01 (0.85-1.19)	1.15 (0.93-1.43)
**Healthcare seeking decision**		<0.001		0.006		
Not involved	385 (18.0)		1			1
Involved	1758 (82.0)		**1.30 (1.08-1.58)**			1.24 (0.99-1.55)
**Age at first birth**		<0.001		0.001		
20 years and above	978 (35.1)		1		1	1
Less than 20 years	1808 (64.9)		**0.79 (0.69-0.90)**		0.92 (0.78-1.07)	0.99 (0.80-1.23)

bold= Significant at p-value <0.05, Secondary model adjusted for residence, provinces, exposure to mass media, working and marital status, level of education, wealth index, timing of ANC initiation, preceding birth interval, age at first sexual intercourse and first birth and healthcare seeking decision making

Details about factors associated with four or more ANC4 contacts (continued care during pregnancy) and four or more ANC contacts and health facility utilization at childbirth (continued care at childbirth) are shown in supplementary files 1 and 2.

### Sensitivity analysis

#### Continued care during pregnancy (Eight or more ANC contacts)

Only 91 (1.2%, 95% CI: 1.0-1.5) women had attended at least eight ANC contacts. In the multivariable analysis, only three factors were associated with eight or more ANC contacts which included: age, residence, and initiation of ANC timing. Younger women aged 15-24 years were 243% (AOR: 3.43 95% CI: 1.46-8.05) more likely to have eight or more ANC contacts compared to their older counterparts aged 35-49 years. Women in urban areas were 245% more likely (AOR: 3.45, 95% CI: 1.48-8.04) to have eight or more ANC contacts compared to women in rural areas. Women who initiated ANC contacts after the first trimester were 92% less likely (AOR: 0.08, 95% CI: 0.03-0.18) to have eight or more ANC contacts compared to women who initiated ANC in the first trimester.

## Discussion

Of the 7,325 women, 38.0% had continuum of maternity care. The level of continued care reduced at every stage of care from 64.1% during pregnancy to 57.3% at child birth and 38% complete continuum of care during postpartum period. The largest dropout occurred between delivery and the postnatal period. This finding of complete continuum of care is lower than findings from Cambodia and Egypt studies [20, 23] and higher than findings from studies conducted in Pakistan, Tanzania, Ethiopia and Ghana [4, 24, 30, 31].

We found that province, timing of ANC initiation, exposure to radio, and the level of education of women and their partners were associated with complete continuum of maternity care. Although preceding birth interval was not associated with complete continuum of care, it was found to be significantly associated with continued care during pregnancy and at childbirth.

Women who initiated ANC after the first trimester were less likely to have complete continuum of care compared to those who initiated ANC in the first trimester. This was observed at all stages of accessing care (continued care during pregnancy and at childbirth). Initiating ANC early enables women to have more ANC contacts thus ability to complete the first continuum which is the first step in ensuring complete continuum of care [32, 33]. The increased likelihood of more ANC contacts associated with early ANC initiation leads to adequate health education sessions regarding the benefits of skilled birth attendance, postnatal care and creates rapport between the health workers and the women [32–34]. This further increases the likelihood of the utilization of the subsequent components of maternity continuum of care. ANC timing has been shown to be associated with complete continuum of care in previous studies [24, 32, 35].

Province was a significant factor at every level of care. However, the association became stronger at the complete continuum of care level. Women belonging to the Western province were less likely to have complete continuum of care compared to the women in the other nine provinces. Variations in the complete continuum of care utilization was large with Lusaka province accounting for the highest percent of women at 17.5% with complete continuum of care, Copperbelt second at 15.1%, Eastern third at 14.4% and Western province with the lowest percentage at 3.9%. This finding is probably related to the availability and ease of access to quality reproductive, maternal and neonatal health services. Yan et al. showed that urban areas of Lusaka and Copperbelt provided maternity services of a higher quality compared to the other regions [16]. Furthermore, projects such as the Saving Mothers, Giving Life (SMGL) initiative implemented in provinces of Eastern and Luapula aimed at addressing the second delay to accessing health care by pregnant women. This increased the proportion of women utilizing health facilities for childbirth between 2012 and 2016 [17]. Since our study included women who had childbirth in the preceding five years of the survey, the impact SMGL programme could in part explain the observed higher likelihood of continuum of care in Luapula and Eastern provinces. Western province women had the least likelihood of complete continuum of care. Western province is characterized by difficult geographical and climatic conditions which has greatly affected agricultural production hence affecting household income [36]. Secondly, over 80% of the population is considered to be poor of which 70% of these are women [36]. These poverty levels can affect access to care hence delayed ANC initiation, access to good quality education and affordability of mass media channels such as radios which factors have been shown to be associated with exposure to the complete continuum of care. This association between the region of residence and continuum of care has be reported in previous studies conducted in Ghana and Cambodia [20, 25].

Exposure to radio was positively associated with continued care during pregnancy and complete continued of care. Women who were exposed to radio were more likely to have more ANC contacts and complete continuum of care. The influence of media on access to maternal healthcare services could be attributed to the role media houses play in reducing knowledge gaps by sensitizing women on the benefits of healthcare seeking and utilization which leads to positive attitudes, challenges negative social norms and improves health seeking behavior [37, 38]. As a result, women exposed to mass media have been shown to be more likely to initiate ANC early in pregnancy and even have more contacts in our analysis and in previous studies [38, 39]. This positive behavior possibly increases their likelihood to have adequate information from the health education sessions which promotes early recognition of danger signs and the need to seek for care from a skilled provider at the earliest opportunity [40]. Furthermore, people who are exposed to media are more likely to be educated, have discussions with others which interpersonal interactions contribute greatly in challenging negative norms that might affect health seeking and hence lead to positive health seeking behavioral change [40, 41]. Exposure to media has been shown in previous studies to have a positive association with continuum of care [4, 39].

Women and those with partners who had attained at least a tertiary level of education were more likely to have complete continuum of care compared to their uneducated counterparts. Sialubanje et al. showed that husbands influenced decisions on the utilisation of health services by pregnant women in Zambia [5]. This is mainly attributed to the centrality of husbands’ roles within the Zambian culture, as family heads and often sole bread winners [5]. Sialubanje et al. also found that availability of funds for birth preparation, presence of a relative as home caretakers and people to work in the field when the woman is away, determined husbands’ decision on whether their wives were to have childbirth from health facilities [5]. Higher levels of education have been shown to be associated with better maternal health literacy, more receptivity to new health related information, increased awareness of available health resources, better decision-making abilities and more financial resources increase their likelihood of accessing maternal healthcare services [32, 42]. With improved maternal health literacy, women and their partners become more informed about maternal health care issues which enables them to make positive health care decisions [43]. In most parts of sub-Saharan Africa including Zambia, the patriarchal system is practiced, hence men have a crucial role in the decision-making process, household resource control and allocation which affects women’s access to healthcare [25]. Education has been shown to help challenge negative norms and lead to better healthcare decision making hence educated men are more likely to support their women to have continuum of care [25]. Therefore, the government of Zambia needs to intensify education to post- secondary level and also improve or start maternal health programs targeting the less educated women and their partners. Education has been shown in previous studies to be associated with continuum of care [4, 25, 32].

### Strengths and limitations

This is the foremost nationwide analysis that explores continuum of maternity care services. Therefore, it can be used as a yardstick and motivation for further studies on related subject matter in order to ensure effective maternal healthcare program implementation. Secondly, we used the most current nationally representative data hence the findings are generalizable to women in Zambia. However, use of cross-sectional data only enables associations to be established but not causal relationships. Sixty-three (0.9%) of the 7325 women could not recall the number of ANC contacts they had and these were categorized among those who discontinued care which might have slightly reduced the percentage of women who had complete continuum of care. Lastly, the possibility of recall bias due to self-reported answers and lack of data on factors such as: distance of residence from health center, availability of emergency obstetric and newborn care services, presence of Safe Motherhood Action Groups, and the role of maternity waiting homes.

## Conclusion

This study demonstrated only two out of five women were able to utilize the entire continuum of maternity care services. Level of education of the women and that of their partners (tertiary), early timing of ANC initiation, residing in other provinces other than the Western province, and exposure to radio were positively associated with completion of maternity continuum of care. Thus, we recommend the different stakeholders engaged in maternal health care to focus on women whose levels of education and that of their partners are below tertiary especially those residing in the Western province to ensure utilization of the entire continuum of maternity care. Utilization of mass media especially radio to sensitize the population on the benefits of continuum of care should be encouraged with great attention given for early initiation of ANC, increasing the number of ANC contacts, health facility and skilled birth attendance during childbirth and postnatal care.

## Supplementary Information

**Additional file 1.**

**Additional file 2.**

## Data Availability

The data set used is publicly available upon permission from MEASURE DHS website (URL: https://www.dhsprogram.com/data/available-datasets.cfm).

## Abbreviations

AOR: Adjusted Odds Ratio
CI: Confidence Interval
COR: Crude Odds Ratio
DHS: Demographic Health Survey
ZDHS: Zambia Demographic Health Survey
OR: Odds Ratio
SD: Standard Deviation
WHO: World Health Organization
SPSS: Statistical Package for Social Science
ANC: Antenatal care
PNC: Post-natal care
