# Supplementary material for: Continuum of maternity care in Zambia: a national representative survey
Source: BMC Pregnancy Childbirth. 2021 Sep 5;21:604. doi: 10.1186/s12884-021-04080-1 (PMC8420052; doi:10.1186/s12884-021-04080-1)
Supplement: Supplementary file 1 — Additional file 1. [file 12884_2021_4080_MOESM1_ESM.docx]

**Continuum of maternity care in Zambia: a national representative survey**

**Authors**

Quraish Sserwanja^1*^, Milton W. Musaba^2^, Linet M. Mutisya^3^, Emmanuel Olal^4^, David Mukunya^5,6^

^1^Programs Department, GOAL, Khartoum, Sudan

Email:qura661@gmail.com

^2^Department of Obstetrics and Gynaecology, Busitema University, Mbale, Uganda

Email: miltonmusaba@gmail.com

^3^Maternal and Child Health Project, Swedish Organization for Global Health, Mayuge, Uganda

Email: [lin4one@gmail.com](mailto:lin4one@gmail.com)

^4^Yotkom Medical Centre, Kitgum, Uganda

Email:e.olal@yahoo.com

^5^Department of Public Health, Busitema University, Mbale, Uganda

Email:zebdaevid@gmail.com

^6^Sanyu Africa Research Institute, Mbale, Uganda

Email:zebdaevid@gmail.com

**^*^Corresponding author**

GOAL

Arkaweet Block 65 House No. 227

Khartoum, Sudan

Tel: +256782295939/+249900933232

Email: [qura661@gmail.com/](mailto:qura661@gmail.com/) [qsserwanja@sd.goal.ie](mailto:qsserwanja@sd.goal.ie)

**Predictors of continued care at pregnancy in Zambia as per ZDHS 2018**

| **Characteristics** | **4 ANC and above n=4651** | **P-value** | **Crude model**  **COR (95%CI)** | **P-value** | **Adjusted Model**  **AOR (95% CI)** |
| --- | --- | --- | --- | --- | --- |
| **Parity** |  | 0.322 |  | 0.457 |  |
| 5 and above | 1310 (28.2) |  | 1 |  |  |
| Less than 5 | 3341 (71.8) |  | 1.06 (0.92-1.22) |  |  |
| **Residence** |  | **<0.001** |  | **0.006** |  |
| Rural | 2946 (63.3) |  | 1 |  | 1 |
| Urban | 1706 (36.7) |  | **0.80 (0.69-0.94)** |  | 0.94 (0.65-1.35) |
| **Provinces** |  | <0.001 |  | 0.001 |  |
| Western | 282 (6.1) |  | 1 |  | 1 |
| Copper belt | 584 (12.6) |  | 0.98 (0.69-1.39) |  | 1.13 (0.70-1.83) |
| Eastern | 639 (13.7) |  | 1.28 (0.93-1.77) |  | 1.34 (0.90-1.99) |
| Luapula | 414 (8.9) |  | 1.23 (0.86-1.78) |  | 1.26 (0.84-1.88) |
| Lusaka | 714 (15.4) |  | 0.92 (0.67-1.28) |  | 1.24 (0.83-1.88) |
| Muchinga | 298 (6.4) |  | **1.42 (1.01-1.98)** |  | 1.36 (0.91-2.01) |
| Northern | 411(8.8) |  | 1.29 (0.82-2.03) |  | 1.57 (0.93-2.65) |
| North Western | 282 (6.1) |  | **1.49 (1.06-2.09)** |  | **2.70 (1.78-4.08)** |
| Southern | 643 (13.8) |  | 1.37 (0.96-1.97) |  | 1.65 (1.00-2.73) |
| Central | 384 (8.3) |  | 0.97 (0.69-1.37) |  | 1.34 (0.88-2.04) |
| **Exposure to Newspapers** |  | 0.057 |  | 0.299 |  |
| No | 3847 (82.7) |  | 1 |  |  |
| Yes | 804 (17.3) |  | 1.14 (0.89-1.44) |  |  |
| **Working status** |  | <0.001 |  | <0.001 |  |
| Not working | 2312 (49.7) |  | 1 |  | 1 |
| Working | 2339 (50.3) |  | **1.34 (1.18-1.53)** |  | 1.17 (0.97-1.40) |
| **Marital status** |  | <0.001 |  | 0.014 |  |
| Not Married | 1092 (23.5) |  | 1 |  |  |
| Married | 3559 (76.5) |  | **1.22 (1.04-1.42)** |  |  |
| **Education Level** |  | <0.001 |  | <0.001 |  |
| No Education | 405 (8.7) |  | 1 |  | 1 |
| Primary Education | 2294 (49.3) |  | **1.25 (1.03-1.53)** |  | 1.15 (0.90-1.47) |
| Secondary Education | 1702 (36.6) |  | 1.15 (0.95-1.41) |  | 1.05 (0.78-1.43) |
| Tertiary | 251 (5.4) |  | **2.75 (1.87-4.07)** |  | 1.83 (0.94-3.56) |
| **Wealth Index** |  | <0.001 |  |  |  |
| Poorest | 1088 (23.4) |  | 1 | 0.155 | 1 |
| Poorer | 993 (21.3) |  | 0.99 (0.84-1.18) |  | 1.03 (0.83-1.28) |
| Middle | 885 (19.0) |  | 0.92 (0.77-1.11) |  | 0.87 (0.68-1.13) |
| Richer | 861 (18.5) |  | 0.74 (0.58-0.94) |  | 0.89 (0.61-1.31) |
| Richest | 826 (17.8) |  | 0.99 (0.80-1.24) |  | 1.00 (0.58-1.72) |
| **Age** |  | 0.128 |  | 0.250 |  |
| 35-49  25-34 | 1108 (23.8)  1925 (41.4) |  | 1  0.94 (0.78-1.12) |  | 1  0.89 (0.72-1.10) |
| 15-24 | 1818 (34.8) |  | 0.88 (0.74-1.04) |  | 0.86 (0.65-1.13) |
| **ANC timing** |  | <0.001 |  |  |  |
| First trimester | 2259 (48.6) |  | 1 | <0.001 | 1 |
| Above first trimester | 2386 (51.4) |  | **0.20 (0.17-0.23)** |  | **0.19 (0.16-0.23)** |
| **Exposure to Radio** |  | <0.001 |  | 0.002 |  |
| No | 2492 (53.6) |  | 1 |  | 1 |
| Yes | 2160 (46.4) |  | **1.22 (1.08-1.38)** |  | **1.23 (1.02-1.49)** |
| **Exposure to TV** |  | 0.076 |  | 0.245 |  |
| No | 3042 (65.4) |  | 1 |  | 1 |
| Yes | 1609 (34.6) |  | 0.91 (0.78-1.06) |  | 0.82 (0.63-1.07) |
| **Preceding Birth Interval** |  | <0.001 |  | <0.001 |  |
| Less than 24 months | 453 (13.0) |  | 1 |  | 1 |
| 24 months and above | 3034 (87.0) | 55  5  66  3f  444  888 | 1.54 (1.29-1.83) |  | **1.55 (1.23-1.94)** |
| **Partner’s education**  **N** | 148 | <0.001 |  | <0.001 |  |
| No education  Primary | 204 (5.9)  1309 |  | 1 |  | 1 |
| Primary | 1292 (37.4)  78  66  66  66 |  | 1.04 (0.79-1.38) |  | 1.05 (0.77-1.44) |
| Secondary | 1611 (46.7) |  | 1.01 (0.76-1.33) |  | 1.15 (0.82-1.63) |
| Tertiary | 344 (10.0) |  | **1.98 (1.32-2.96)** |  | **1.87 (1.08-3.26)** |
| **Age at first sex** |  | 0.029 |  | 0.088 |  |
| 18 and above | 1236 (26.6) |  | 1 |  | 1 |
| Less than 18 | 3415 (73.4) |  | 0.89 (0.77-1.02) |  | 1.09 (0.88-1.35) |
| **Healthcare seeking decision^d^** |  | 0.791 |  | 0.834 |  |
| Not involved | 732 (20.6) |  | 1 |  |  |
| Involved | 2827 (79.4) |  | 1.02 (0.86-1.21) |  |  |
| **Age at first birth** |  | 0.012 |  | 0.108 |  |
| 20 and above | 1533 (33.0) |  | 1 |  | 1 |
| Less than 20 | 3118 (67.0) |  | 0.88 (0.75-1.03) |  | 1.06 (0.83-1.35) |

**Bold** significant at p-value less than 0.05
