# Supplementary material for: Continuum of maternity care in Zambia: a national representative survey
Source: BMC Pregnancy Childbirth. 2021 Sep 5;21:604. doi: 10.1186/s12884-021-04080-1 (PMC8420052; doi:10.1186/s12884-021-04080-1)
Supplement: Supplementary file 2 — Additional file 2. [file 12884_2021_4080_MOESM2_ESM.docx]

**Continuum of maternity care in Zambia: a national representative survey**

**Authors**

Quraish Sserwanja^1*^, Milton W. Musaba^2^, Linet M. Mutisya^3^, Emmanuel Olal^4^, David Mukunya^5,6^

^1^Programs Department, GOAL, Khartoum, Sudan

Email:qura661@gmail.com

^2^Department of Obstetrics and Gynaecology, Busitema University, Mbale, Uganda

Email: miltonmusaba@gmail.com

^3^Maternal and Child Health Project, Swedish Organization for Global Health, Mayuge, Uganda

Email: [lin4one@gmail.com](mailto:lin4one@gmail.com)

^4^Yotkom Medical Centre, Kitgum, Uganda

Email:e.olal@yahoo.com

^5^Department of Public Health, Busitema University, Mbale, Uganda

Email:zebdaevid@gmail.com

^6^Sanyu Africa Research Institute, Mbale, Uganda

Email:zebdaevid@gmail.com

**^*^Corresponding author**

GOAL

Arkaweet Block 65 House No. 227

Khartoum, Sudan

Tel: +256782295939/+249900933232

Email: [qura661@gmail.com/](mailto:qura661@gmail.com/) [qsserwanja@sd.goal.ie](mailto:qsserwanja@sd.goal.ie)

**Predictors of continued care at childbirth in Zambia**

| **Characteristics** | **4 ANC+ and facility delivery n=4199** | **P-value** | **Crude model**  **COR (95%CI)** | **P-value** | **Adjusted Model**  **AOR (95% CI)** |
| --- | --- | --- | --- | --- | --- |
| **Parity** |  | <0.001 |  | 0.001 |  |
| 5 and above | 1112 (26.5) |  | 1 |  | 1 |
| Less than 5 | 3087 (73.5) |  | **1.27 (1.11-1.45)** |  | 1.10 (0.94-1.30) |
| **Residence** |  | 0.308 |  | 0.558 |  |
| Rural | 2566 (61.1) |  | 1 |  |  |
| Urban | 1632 (38.9) |  | 1.05 (0.89-1.24) |  |  |
| **Provinces** |  | <0.001 |  | 0.001 |  |
| Western | 241 (5.7) |  | 1 |  | 1 |
| Copperbelt | 545 (13.0) |  | 1.26 (0.90-1.77) |  | 1.09 (0.68-1.76) |
| Eastern | 608 (14.5) |  | **1.59 (1.18-2.15)** |  | **1.76 (1.20-2.59)** |
| Luapula | 387 (9.2) |  | **1.50 (1.06-2.12**) |  | 1.51 (0.99-2.29) |
| Lusaka | 673 (16.0) |  | 1.21 (0.88-1.64) |  | 1.05 (0.69-1.58) |
| Muchinga | 253 (6.0) |  | 1.38 (0.93-2.05) |  | 1.32 (0.84-2.09) |
| Northern | 350 (8.3) |  | 1.29 (0.81-2.07) |  | 1.47 (0.85-2.54) |
| North Western | 255 (6.1) |  | **1.68 (1.21-2.33)** |  | **2.27 (1.49-3.47)** |
| Southern | 571 (13.6) |  | **1.49 (1.01-2.21)** |  | 1.56 (0.94-2.59) |
| Central | 314 (7.5) |  | 0.94 (0.67-1.33) |  | 0.97 (0.64-1.47) |
| **Exposure to Newspapers** |  | <0.001 |  | 0.010 |  |
| No | 3427 (81.6) |  | 1 |  | 1 |
| Yes | 771 (18.4) |  | **1.36 (1.07-1.71)** |  | 1.08 (0.84-1.38) |
| **Working status** |  | <0.001 |  | 0.001 |  |
| Not working | 2100 (50.0) |  | 1 |  | 1 |
| Working | 2098 (50.0) |  | **1.24 (1.09-1.42)** |  | 1.07 (0.89-1.28) |
| **Marital status** |  | 0.004 |  | 0.038 |  |
| Not Married | 990 (23.6) |  | 1 |  | - |
| Married | 3209 (76.4) |  | **1.17 (1.01-1.36)** |  | - |
| **Education Level** |  | <0.001 |  | <0.001 |  |
| No Education | 322 (7.7) |  | 1 |  | 1 |
| Primary Education | 2002 (47.7) |  | **1.43 (1.16-1.76)** |  | 1.24 (0.96-1.61) |
| Secondary Education | 1624 (38.7) |  | **1.68 (1.35-2.09)** |  | 1.22 (0.89-4.67) |
| Tertiary | 250 (6.0) |  | **4.34 (2.92-6.46)** |  | **2.07 (1.06-4.05)** |
| **Wealth Index** |  | <0.001 |  | 0.007 |  |
| Poorest | 913 (21.7) |  | 1 |  | 1 |
| Poorer | 868 (20.7) |  | 1.10 (0.93-1.31) |  | 1.16 (0.93-1.44) |
| Middle | 794 (18.9) |  | 1.11 (0.93-1.33) |  | 1.05 (0.82-1.34) |
| Richer | 809 (19.3) |  | 1.02 (0.80-1.31) |  | 1.17 (0.78-1.74) |
| Richest | 815 (19.4) |  | **1.52 (1.22-1.90)** |  | 1.35 (0.89-2.03) |
| **Age** |  | 0.943 |  | 0.969 |  |
| 35-49  25-34 | 968 (23.1)  1736 (41.3) |  | 1  1.02 (0.86-1.22) |  |  |
| 15-24 | 1495 (35.6) |  | 1.02 (0.87-1.19) |  |  |
| **ANC timing** |  | <0.001 |  | <0.001 |  |
| First trimester | 2062 (49.2) |  | 1 |  | 1 |
| Above first trimester | 2131 (50.8) |  | **0.27 (0.24-0.30)** |  | **0.27 (0.23-0.31)** |
| **Exposure to Radio** |  | <0.001 |  | <0.001 |  |
| No | 2195 (52.3) |  | 1 |  | 1 |
| Yes | 2003 (47.7) |  | **1.32 (1.18-1.49)** |  | 1.18 (0.99-1.41) |
| **Exposure to TV** |  | 0.002 |  | 0.046 |  |
| No | 2658 (63.3) |  | 1 |  | 1 |
| Yes | 1541 (36.7) |  | **1.16 (1.01-1.35**) |  | 0.91 (0.72-1.15) |
| **Preceding Birth Interval** |  | <0.001 |  | <0.001 |  |
| Less than 24 months | 380 (12.3) |  | 1 |  | 1 |
| 24 months and above | 2706 (87.7) | 55  5  66  3f  444  888 | **1.61 (1.36-1.90)** |  | **1.57 (1.27-1.94)** |
| **Partner’s education**  **N** | 148 | <0.001 |  | <0.001 |  |
| No education  Primary | 167 (5.4)  1309 |  | 1 |  | 1 |
| Primary | 1106 (35.5)  78  66  66  66 |  | 1.13 (0.86-1.50) |  | 1.13 (0.83-1.53) |
| Secondary | 1498 (48.1) |  | **1.35 (1.02-1.77)** |  | 1.33 (0.96-1.84) |
| Tertiary | 341 (11.0) |  | **3.07 (2.05-4.60)** |  | **2.25 (1.33-3.83)** |
| **Age at first sex** |  | <0.001 |  | <0.001 |  |
| 18 and above | 1166 (27.8) |  | 1 |  | 1 |
| Less than 18 | 3033 (72.2) |  | **0.77 (0.67-0.89)** |  | 1.03 (0.84-1.26) |
| **Healthcare seeking decision^d^** |  | 0.053 |  | 0.111 |  |
| Not involved | 631 (19.7) |  | 1 |  | 1 |
| Involved | 2577 (80.3) |  | 1.14 (0.97-1.34) |  | 1.18 (0.97-1.43) |
| **Age at first birth** |  | <0.001 |  | 0.009 |  |
| 20 and above | 1422 (33.9) |  | 1 |  | 1 |
| Less than 20 | 2777 (66.1) |  | **0.81 (0.70-0.95)** |  | 1.08 (0.86-1.36) |

**Bold** significant at p-value less than 0.05
